# Poly[μ_2_-hydroxido-μ_4_-sulfato-neodym­ium(III)]

**DOI:** 10.1107/S1600536808021818

**Published:** 2008-07-19

**Authors:** Tao Zhang, Jingmei Lu

**Affiliations:** a School of Life Sciences, Northeast Normal University, Changchun 130024, People’s Republic of China

## Abstract

The title compound, [Nd(OH)(SO_4_)]_*n*_, was obtained hydro­thermally from an aqueous solution of neodymium nitrate, 1,2-propane­diamine and sulfuric acid. The structure features nona­coordinated neodymium with sulfate and hydroxide anions acting as bridging ligands. The OH group forms a weak O—H⋯O hydrogen bond with an O⋯O distance of 3.224 (5) Å.

## Related literature

For related literature, see: Doran *et al.* (2002 ▶); Xu, Ding, Zhou & Liu (2006 ▶); Xu, Ding, Feng *et al.* (2006 ▶); Xu *et al.* (2007 ▶); Yuan *et al.* (2004 ▶); Zhang *et al.* (2004 ▶); Ding *et al.* (2006 ▶).

## Experimental

### 

#### Crystal data

[Nd(OH)(SO_4_)]
*M* *_r_* = 257.31
Monoclinic, 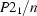

*a* = 4.4678 (9) Å
*b* = 12.432 (2) Å
*c* = 6.8575 (13) Å
β = 106.324 (3)°
*V* = 365.53 (12) Å^3^
*Z* = 4
Mo *K*α radiation
μ = 14.66 mm^−1^
*T* = 293 (2) K
0.10 × 0.08 × 0.06 mm

#### Data collection

Bruker APEXII CCD diffractometer
Absorption correction: multi-scan (*SADABS*; Sheldrick, 2003 ▶) *T*_min_ = 0.322, *T*_max_ = 0.473 (expected range = 0.282–0.415)
1837 measured reflections
675 independent reflections
669 reflections with *I* > 2σ(*I*)
*R*_int_ = 0.013

#### Refinement

*R*[*F* ^2^ > 2σ(*F* ^2^)] = 0.022
*wR*(*F* ^2^) = 0.056
*S* = 1.24
675 reflections
67 parameters
1 restraint
H atoms treated by a mixture of independent and constrained refinement
Δρ_max_ = 0.56 e Å^−3^
Δρ_min_ = −2.27 e Å^−3^

### 

Data collection: *APEX2* (Bruker, 2005 ▶); cell refinement: *SAINT* (Bruker, 2005 ▶); data reduction: *SAINT*; program(s) used to solve structure: *SHELXS97* (Sheldrick, 2008 ▶); program(s) used to refine structure: *SHELXL97* (Sheldrick, 2008 ▶); molecular graphics: *SHELXTL* (Sheldrick, 2008 ▶); software used to prepare material for publication: *SHELXTL*.

## Supplementary Material

Crystal structure: contains datablocks global, I. DOI: 10.1107/S1600536808021818/br2079sup1.cif

Structure factors: contains datablocks I. DOI: 10.1107/S1600536808021818/br2079Isup2.hkl

Additional supplementary materials:  crystallographic information; 3D view; checkCIF report

## Figures and Tables

**Table 1:** Hydrogen-bond geometry (Å, °)

*D*—H⋯*A*	*D*—H	H⋯*A*	*D*⋯*A*	*D*—H⋯*A*
O5—H1⋯O1^i^	0.83 (3)	2.43 (3)	3.224 (5)	160 (6)

Symmetry code: (i) 
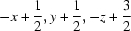
.

**Fig. 1.**
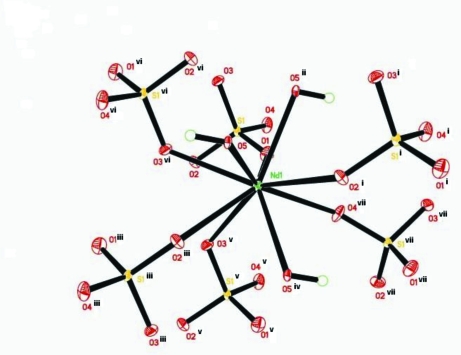
The molecular structure for title compound. Displacement ellipsoids at the 50% probability level.

**Fig. 2.**
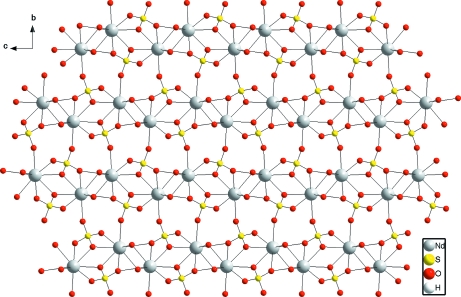
The crystal packing in the unit cell of Nd(SO4)(OH).

## supplementary crystallographic information

### Comment

In the last few years, the synthesis of new three dimensional lanthanide sulfates have received great attention, due to their functional applications in catalysis, ion-exchange, and optical device (Zhang *et al.*,2004; Yuan *et al.*, 2004; Xu, Ding, Feng *et al.*, 2006; Xu, Ding, Zhou & Liu, 2006; Doran *et al.*, 2002, Xu *et al.*, 2007). In this work, we designed and synthesized the title compound, neodymium(3+) sulfate hydroxide, which features a three–dimensional framework constructed from NdO_9_ polyhedra and SO_4_ tetrahedra.

Nd(SO_4_)(OH) is isostructural with La(SO_4_)(OH) (Zhang *et al.*,2004) and Eu(SO_4_)(OH)(Ding *et al.*,2006), the framework of title compound constructed from NdO_9_ polyhedra and SO_4_ tetrahedra. As show in Fig. 1, the asymmetric unit contains one Nd^3+^, one SO_4_^2–^ group and one hydroxide group. The Nd^3+^ is coordinated by six bridging sulfate ions, each S atom makes four S–O–Nd linkages by sharing the bridging O atoms. The coordination sphere of Nd is completed by three OH^-^ groups, which act as briding ligands between three Nd^3+^.

The O-H group is involved hydrogen bonding interactions with O1, O2 and O4, the distances of O—H···O are vary from 2.60 (2) to 2.90 (2) Å.

The Nd —O distances are between of 2.374 (4)– 2.800 (4)Å (Table 1)while the O—Nd—O angles are between 66.02 (13) and 141.55 (12)°. These bond distances and bond angles are in agreement with those found in the reported rare-earth compounds (Zhang *et al.*,2004; Ding *et al.*,2006). The bond distances of S—O and angles of O—S—O are unexceptional. Fig. 2 shows the three-dimensional arrangement in the unit cell, displaying the way the different Nd^3+^ are connected by bridging hydroxide and sulfates groups.

### Experimental

Pink block crystals were synthesized hydrothermally from a mixture of Nd(NO_3_)_3_.6H_2_O, 1,2-propane diamine, H_2_SO_4_ and water. In a typical synthesis, Nd(NO_3_)_3_.6H_2_O (0.6066 g) was dissolved in a mixture of 1,2-propane diamine (0.2205 g) and water (3.2 ml) followed by the addition of H_2_SO_4_ (98%) (0.3093 g) with constant stirring. Finally, the mixture was kept in a 25 ml Teflon-lined steel autoclave at 180 °C for 7 days. After the autoclave was slowly cooled to room temperature, Pink block crystals of the title compound were obtained.

### Refinement

The H atom of water was located from difference map, while the distance of O—H was restrained as 0.85 (2) Å.

### Crystal data

**Table:**

[Nd(OH)(SO_4_)]	*F*_000_ = 468
*M**_r_* = 257.31	*D*_x_ = 4.676 Mg m^−^^3^
Monoclinic, *P*2_1_/*n*	Mo *K*α radiation λ = 0.71073 Å
Hall symbol: -P 2yn	Cell parameters from 150 reflections
*a* = 4.4678 (9) Å	θ = 2.3–22.5º
*b* = 12.432 (2) Å	µ = 14.66 mm^−^^1^
*c* = 6.8575 (13) Å	*T* = 293 (2) K
β = 106.324 (3)º	Block, pink
*V* = 365.53 (12) Å^3^	0.10 × 0.08 × 0.06 mm
*Z* = 4	

### Data collection

**Table:**

Bruker APEXII CCD diffractometer	675 independent reflections
Radiation source: fine-focus sealed tube	669 reflections with *I* > 2σ(*I*)
Monochromator: graphite	*R*_int_ = 0.013
*T* = 293(2) K	θ_max_ = 25.5º
ω scans	θ_min_ = 3.3º
Absorption correction: multi-scan(SADABS; Sheldrick, 2003)	*h* = −3→5
*T*_min_ = 0.322, *T*_max_ = 0.473	*k* = −14→15
1837 measured reflections	*l* = −7→8

### Refinement

**Table:**

Refinement on *F*^2^	Secondary atom site location: difference Fourier map
Least-squares matrix: full	Hydrogen site location: difference Fourier map
*R*[*F*^2^ > 2σ(*F*^2^)] = 0.022	H atoms treated by a mixture of independent and constrained refinement
*wR*(*F*^2^) = 0.056	*w* = 1/[σ^2^(*F*_o_^2^) + (0.035*P*)^2^ + 0.7631*P*] where *P* = (*F*_o_^2^ + 2*F*_c_^2^)/3
*S* = 1.24	(Δ/σ)_max_ = 0.001
675 reflections	Δρ_max_ = 0.56 e Å^−^^3^
67 parameters	Δρ_min_ = −2.27 e Å^−^^3^
1 restraint	Extinction correction: none
Primary atom site location: structure-invariant direct methods	

### Special details

**Table:**

Geometry. All e.s.d.'s (except the e.s.d. in the dihedral angle between two l.s. planes) are estimated using the full covariance matrix. The cell e.s.d.'s are taken into account individually in the estimation of e.s.d.'s in distances, angles and torsion angles; correlations between e.s.d.'s in cell parameters are only used when they are defined by crystal symmetry. An approximate (isotropic) treatment of cell e.s.d.'s is used for estimating e.s.d.'s involving l.s. planes.
Refinement. Refinement of *F*^2^ against ALL reflections. The weighted *R*-factor *wR* and goodness of fit *S* are based on *F*^2^, conventional *R*-factors *R* are based on *F*, with *F* set to zero for negative *F*^2^. The threshold expression of *F*^2^ > σ(*F*^2^) is used only for calculating *R*-factors(gt) *etc*. and is not relevant to the choice of reflections for refinement. *R*-factors based on *F*^2^ are statistically about twice as large as those based on *F*, and *R*- factors based on ALL data will be even larger.

### Fractional atomic coordinates and isotropic or equivalent isotropic displacement parameters (Å^2^)

**Table:**

	*x*	*y*	*z*	*U*_iso_*/*U*_eq_	
Nd1	0.14116 (6)	0.93569 (2)	0.80136 (4)	0.00655 (15)	
S1	0.4852 (3)	0.85400 (10)	0.38900 (18)	0.0059 (3)	
O1	0.3672 (9)	0.8343 (3)	0.5628 (6)	0.0136 (8)	
O2	0.2485 (9)	0.9040 (3)	0.2196 (6)	0.0127 (8)	
O3	0.7563 (9)	0.9295 (3)	0.4498 (6)	0.0105 (8)	
O4	0.5923 (9)	0.7539 (3)	0.3200 (6)	0.0129 (8)	
O5	0.3028 (9)	1.0847 (3)	1.0385 (6)	0.0081 (7)	
H1	0.295 (14)	1.148 (2)	0.997 (9)	0.010*	

### Atomic displacement parameters (Å^2^)

**Table:**

	*U*^11^	*U*^22^	*U*^33^	*U*^12^	*U*^13^	*U*^23^
Nd1	0.0075 (2)	0.0051 (2)	0.0073 (2)	0.00001 (8)	0.00245 (14)	−0.00073 (8)
S1	0.0073 (6)	0.0040 (6)	0.0068 (6)	0.0003 (4)	0.0026 (5)	−0.0001 (4)
O1	0.0172 (19)	0.0129 (19)	0.0136 (19)	0.0013 (16)	0.0093 (16)	0.0003 (15)
O2	0.0096 (18)	0.0133 (18)	0.0131 (18)	0.0026 (16)	−0.0002 (15)	0.0032 (15)
O3	0.0088 (19)	0.008 (2)	0.015 (2)	−0.0023 (13)	0.0036 (16)	−0.0004 (13)
O4	0.0196 (19)	0.0066 (19)	0.015 (2)	0.0037 (15)	0.0092 (17)	−0.0002 (14)
O5	0.0080 (18)	0.0034 (16)	0.0122 (18)	0.0005 (14)	0.0016 (14)	0.0027 (14)

### Geometric parameters (Å, °)

**Table:**

Nd1—O4^i^	2.374 (4)	S1—O1	1.453 (4)
Nd1—O5^ii^	2.431 (4)	S1—O4	1.459 (4)
Nd1—O5	2.437 (4)	S1—O2	1.470 (4)
Nd1—O1	2.492 (4)	S1—O3	1.496 (4)
Nd1—O3^iii^	2.535 (4)	O2—Nd1^vi^	2.624 (4)
Nd1—O5^iv^	2.536 (4)	O2—Nd1^viii^	2.800 (4)
Nd1—O3^v^	2.538 (4)	O3—Nd1^iii^	2.535 (4)
Nd1—O2^vi^	2.624 (4)	O3—Nd1^ix^	2.538 (4)
Nd1—O2^vii^	2.800 (4)	O4—Nd1^x^	2.374 (4)
Nd1—Nd1^iv^	3.6744 (7)	O5—Nd1^ii^	2.431 (4)
Nd1—Nd1^ii^	3.9178 (7)	O5—Nd1^iv^	2.536 (4)
			
O4^i^—Nd1—O5^ii^	88.26 (13)	O4^i^—Nd1—Nd1^iv^	109.65 (10)
O4^i^—Nd1—O5	137.19 (13)	O5^ii^—Nd1—Nd1^iv^	103.34 (9)
O5^ii^—Nd1—O5	72.81 (14)	O5—Nd1—Nd1^iv^	43.41 (9)
O4^i^—Nd1—O1	66.02 (13)	O1—Nd1—Nd1^iv^	173.45 (9)
O5^ii^—Nd1—O1	72.10 (13)	O3^iii^—Nd1—Nd1^iv^	112.42 (8)
O5—Nd1—O1	136.35 (13)	O5^iv^—Nd1—Nd1^iv^	41.33 (8)
O4^i^—Nd1—O3^iii^	136.85 (12)	O3^v^—Nd1—Nd1^iv^	115.79 (9)
O5^ii^—Nd1—O3^iii^	91.10 (13)	O2^vi^—Nd1—Nd1^iv^	49.41 (8)
O5—Nd1—O3^iii^	82.80 (13)	O2^vii^—Nd1—Nd1^iv^	45.37 (8)
O1—Nd1—O3^iii^	72.80 (12)	O4^i^—Nd1—Nd1^ii^	115.94 (9)
O4^i^—Nd1—O5^iv^	77.43 (13)	O5^ii^—Nd1—Nd1^ii^	36.45 (9)
O5^ii^—Nd1—O5^iv^	128.19 (16)	O5—Nd1—Nd1^ii^	36.35 (9)
O5—Nd1—O5^iv^	84.74 (13)	O1—Nd1—Nd1^ii^	105.03 (9)
O1—Nd1—O5^iv^	138.09 (13)	O3^iii^—Nd1—Nd1^ii^	86.21 (9)
O3^iii^—Nd1—O5^iv^	132.32 (12)	O5^iv^—Nd1—Nd1^ii^	109.06 (9)
O4^i^—Nd1—O3^v^	88.46 (12)	O3^v^—Nd1—Nd1^ii^	151.19 (8)
O5^ii^—Nd1—O3^v^	139.42 (13)	O2^vi^—Nd1—Nd1^ii^	97.41 (9)
O5—Nd1—O3^v^	130.63 (11)	O2^vii^—Nd1—Nd1^ii^	58.27 (8)
O1—Nd1—O3^v^	69.68 (13)	Nd1^iv^—Nd1—Nd1^ii^	72.017 (16)
O3^iii^—Nd1—O3^v^	65.06 (14)	O1—S1—O4	110.5 (2)
O5^iv^—Nd1—O3^v^	90.27 (13)	O1—S1—O2	111.9 (2)
O4^i^—Nd1—O2^vi^	133.45 (13)	O4—S1—O2	109.4 (2)
O5^ii^—Nd1—O2^vi^	133.02 (12)	O1—S1—O3	109.2 (2)
O5—Nd1—O2^vi^	61.74 (12)	O4—S1—O3	108.2 (2)
O1—Nd1—O2^vi^	137.14 (12)	O2—S1—O3	107.5 (2)
O3^iii^—Nd1—O2^vi^	72.81 (13)	S1—O1—Nd1	139.5 (2)
O5^iv^—Nd1—O2^vi^	60.80 (12)	S1—O2—Nd1^vi^	133.1 (2)
O3^v^—Nd1—O2^vi^	73.07 (12)	S1—O2—Nd1^viii^	138.3 (2)
O4^i^—Nd1—O2^vii^	78.28 (12)	Nd1^vi^—O2—Nd1^viii^	85.22 (11)
O5^ii^—Nd1—O2^vii^	70.32 (12)	S1—O3—Nd1^iii^	120.8 (2)
O5—Nd1—O2^vii^	59.36 (12)	S1—O3—Nd1^ix^	124.3 (2)
O1—Nd1—O2^vii^	128.08 (12)	Nd1^iii^—O3—Nd1^ix^	114.94 (14)
O3^iii^—Nd1—O2^vii^	141.03 (12)	S1—O4—Nd1^x^	155.2 (3)
O5^iv^—Nd1—O2^vii^	58.11 (12)	Nd1^ii^—O5—Nd1	107.19 (14)
O3^v^—Nd1—O2^vii^	147.55 (12)	Nd1^ii^—O5—Nd1^iv^	128.19 (16)
O2^vi^—Nd1—O2^vii^	94.78 (11)	Nd1—O5—Nd1^iv^	95.26 (12)

Symmetry codes: (i) *x*−1/2, −*y*+3/2, *z*+1/2; (ii) −*x*+1, −*y*+2, −*z*+2; (iii) −*x*+1, −*y*+2, −*z*+1; (iv) −*x*, −*y*+2, −*z*+2; (v) *x*−1, *y*, *z*; (vi) −*x*, −*y*+2, −*z*+1; (vii) *x*, *y*, *z*+1; (viii) *x*, *y*, *z*−1; (ix) *x*+1, *y*, *z*; (x) *x*+1/2, −*y*+3/2, *z*−1/2.

### Hydrogen-bond geometry (Å, °)

**Table:**

*D*—H···*A*	*D*—H	H···*A*	*D*···*A*	*D*—H···*A*
O5—H1···O1^xi^	0.83 (3)	2.43 (3)	3.224 (5)	160 (6)

Symmetry codes: (xi) −*x*+1/2, *y*+1/2, −*z*+3/2.
